# *Salmo salar *and *Esox lucius *full-length cDNA sequences reveal changes in evolutionary pressures on a post-tetraploidization genome

**DOI:** 10.1186/1471-2164-11-279

**Published:** 2010-04-30

**Authors:** Jong S Leong, Stuart G Jantzen, Kristian R von Schalburg, Glenn A Cooper, Amber M Messmer, Nancy Y Liao, Sarah Munro, Richard Moore, Robert A Holt, Steven JM Jones, William S Davidson, Ben F Koop

**Affiliations:** 1 Biology, Centre for Biomedical Research, University of Victoria, Victoria, British Columbia, V8W 3N5 Canada; 2 Genome Sciences Centre, BC Cancer Agency, Vancouver, British Columbia, V5Z 4E6 Canada; 3 Molecular Biology and Biochemistry, Simon Fraser University, Burnaby, British Columbia, V5A 1S6 Canada

## Abstract

**Background:**

Salmonids are one of the most intensely studied fish, in part due to their economic and environmental importance, and in part due to a recent whole genome duplication in the common ancestor of salmonids. This duplication greatly impacts species diversification, functional specialization, and adaptation. Extensive new genomic resources have recently become available for Atlantic salmon (*Salmo salar*), but documentation of allelic versus duplicate reference genes remains a major uncertainty in the complete characterization of its genome and its evolution.

**Results:**

From existing expressed sequence tag (EST) resources and three new full-length cDNA libraries, 9,057 reference quality full-length gene insert clones were identified for Atlantic salmon. A further 1,365 reference full-length clones were annotated from 29,221 northern pike (*Esox lucius*) ESTs. Pairwise d_N_/d_S _comparisons within each of 408 sets of duplicated salmon genes using northern pike as a diploid out-group show asymmetric relaxation of selection on salmon duplicates.

**Conclusions:**

9,057 full-length reference genes were characterized in *S. salar *and can be used to identify alleles and gene family members. Comparisons of duplicated genes show that while purifying selection is the predominant force acting on both duplicates, consistent with retention of functionality in both copies, some relaxation of pressure on gene duplicates can be identified. In addition, there is evidence that evolution has acted asymmetrically on paralogs, allowing one of the pair to diverge at a faster rate.

## Background

Salmonidae (including salmon, trout, charr, whitefish and grayling) are of economic and environmental importance, leading to a high level of interest in many different areas of biology. Of the sixty-six species in this family [1], Atlantic salmon (*Salmo salar*) has been used as a model for studies in several areas including osmoregulation, environmental toxicology, immunology, growth, physiology, and genomics [2-25]. Both *S. salar *and the closely related rainbow trout (*Oncorhynchus mykiss*) are commonly used as important sentinel species to monitor the health of aquatic environments [26]. Conservation and enhancement of wild stocks of these fish continues to be the subject of very large internationally concerned groups [27,28]. Basic biological knowledge of *S. salar *serves as a foundation for improving fish health, conserving wild stocks, and increasing the commercial sustainability of aquaculture. Recent efforts in genomics have provided new tools to address fundamental questions regarding fish health, ecology, physiology, and genetics, as well as allowing investigation of post-tetraploidization genome remodelling [29-34]. Detailed efforts to annotate the entire complement of *S. salar *genes will greatly facilitate a better understanding of all aspects of salmonid biology.

The study of salmonid genomes is made more difficult and biologically interesting because of a whole genome duplication (WGD) that occurred through an autotetraploidization event in the common ancestor of salmonids between 25-100 million years ago [35]. Extant salmonids are currently in a pseudotetraploid state and are in the process of reverting to a stable diploid state [32]. Though many of the gene duplicates from the WGD have been lost through deletion events or by being converted into pseudogenes, many sets of paralogs remain. As a result, there are practical problems in distinguishing among alleles, recent segmental duplications, gene family members, and duplications arising from the WGD. Experimentally, *S. salar *genes have proven challenging to characterize because of the complexities resulting from assembling large numbers of partial mRNA sequences represented by expressed sequence tags (ESTs) obtained from these duplicated and other closely related sequences. In addition, interspersed repeat sequences [36] can lead to the formation of incorrect assemblies of genomic sequences and transcripts (contigs). To resolve these potential errors, a gene containing coding sequence (CDS) flanked by 5' and 3' untranslated regions (UTR) coming from a single, completely characterized cDNA clone provides an important reference sequence representing a single allele of a single gene. The expansion of reference clone resources are particularly important not only in identifying other potential alleles and gene duplicates that are so pervasive in the pseudotetraploid salmonids, but also in studying fundamental genetic rates and modes of evolutionary change.

Relatively few organisms and lineages have been used to examine the evolution of duplicated genes following a WGD. Morin et al. [37] investigated the selective pressures acting on paralogs in *Xenopus laevis*, which resulted from allotetraploidization, and found wide-spread purifying selection but with some relaxation of pressure relative to orthologs in the diploid *Xenopus tropicalis*. Maere et al. [38] studied substitution rates and found certain functional categories of genes that were selectively lost after genome duplication events in *Arabidopsis thaliana*. In a larger scale study, Conant and Wagner [39] researched the genomes of a number of different organisms that have undergone WGDs, testing for asymmetric divergence of paralogs which they found in 20 - 30% of duplicates. Looking at asymmetrically evolving paralogs in yeast, Turunen et al. [40] recently presented evidence for relaxation of selective pressures. Furthermore, in another examination of the WGD in *S. cerevisiae*, positive selection was detected in a substantial portion of paralogs [41]. These studies examined the ratio of amino acid changing substitutions to silent substitutions (d_N_/d_S_) to measure evolutionary rates. The present study incorporates some of these approaches to identify evolutionary patterns in the genome of *S. salar*. Since there is not a large number of examples of post-tetraploidization evolution available for study, the WGD in salmonids becomes an important area for research.

Of the few organisms studied, some have been examined by a number of research groups. Since this is one of the first studies examining the post-tetraploidization evolutionary patterns in the salmonid genome, it is our hope that other groups in addition to our own will expand on the work presented here, incorporating growing datasets and using a wide variety of phylogenetic and evolutionary methods.

Characterizing evolutionary changes in polyploid genomes requires comparison to a pre-WGD out-group species so that differences in substitution rates with respect to an ancestral genomic state can be determined. Ishiguro et al., Lopez et al., and Li et al. [42-44] report that the Order Esociformes is the closest non-polyploid sister group to the Salmoniformes. Karyotypic data [45,46] and C-values of ~3.0 - 3.3 pg in salmonids and ~0.9 - 1.4 pg in esocids [47] are consistent with the occurrence of the WGD after the divergence of esocids and salmonids. In particular, studying northern pike (*Esox lucius*) as a representative of the order would provide an opportunity to continue building upon existing efforts. As there were only 158 core nucleotide sequences, 83 protein sequences, and 3,612 EST sequences [33] available for northern pike prior to this study, it was necessary to expand sequence information of this species before a more thorough analysis of salmonid gene duplications could be done.

The objectives of this study were to: 1) obtain a large number of full-length reference cDNA clone sequences; 2) expand the transcriptomic resources (ESTs) of *E. lucius; *and 3) identify evolutionary patterns of duplicated genes in the autotetraploid *S. salar *species.

## Results

### Full-length cDNA library construction and analysis

The majority of existing EST data from *S. salar *came from highly normalized cDNA libraries that were full-length (FL) biased [31,33,48,49]. To specifically identify more full-length transcripts, a protocol for enrichment of 5'-CAPed mRNA was employed which prevents truncated mRNA from being reverse-transcribed, followed by transfer of intact double-stranded cDNAs directly into the library vector using Invitrogen Gateway^® ^recombination cloning [50]. Starting from *S. salar *brain, head kidney, and spleen tissues, three non-normalized, size selected, full-length libraries were constructed. mRNAs were size-selected for 600 to 1,100 bp (rgg), 1,100 to 2,000 bp (rgh) and >2,200 bp (rgf). 7,680, 7,680, and 16,128 clones from rgg, rgh, and rgf, respectively, were bi-directionally sequenced. For the short insert library (rgg), 11,917 sequences were obtained and assembled into 1,833 transcripts (903 singletons and 930 contigs). This library had the fewest novel transcripts and had the highest redundancy in terms of identified sequences. The majority of transcripts in this library were identified as hemoglobins, ribosomal protein genes or other genes previously seen in our existing EST dataset [33]. For the mid-sized insert library (rgh), 12,250 sequences were obtained and assembled into 5,305 transcripts (3,088 singletons and 2,217 contigs). While the sequence diversity of this library was higher, nearly all complete transcripts had been previously identified [33]. For the large-sized insert library (rgf), 30,415 sequences were obtained and assembled into 15,125 transcripts (11,190 singletons and 3,935 contigs). This library contained the highest number of novel transcripts.

### Identification of *S. salar *Full-Length cDNA contigs from existing EST assemblies

**Figure 1:**
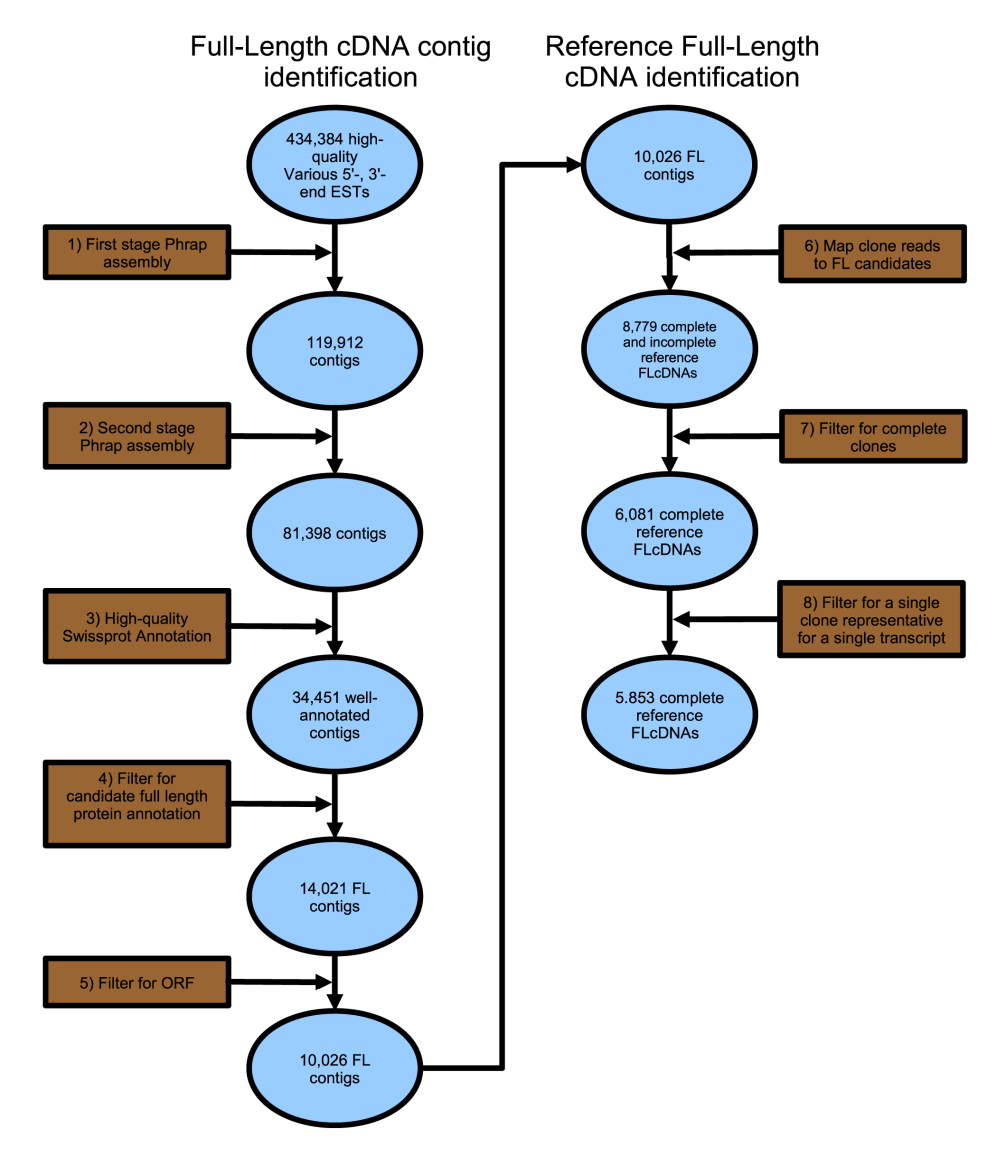
**Schematic of *S. salar *FLcDNA contig identification and reference FLcDNA identification**. Two-stage assembly of 434,384 high-quality 5'- and 3'-end ESTs identified 81,398 contigs (1-2) for FL contig identification. A BLASTX was carried out resulting in 34,451 well-annotated contigs (3), which were further reduced to 14,021 FL annotations by increasing the stringency of the local alignment length (4). In-frame annotation-flanking start and stop codons were found from the reduced set, resulting in a set of 10,026 FL contigs (5). The FL contigs represent the complete set of FL unique putative transcripts. A set of all reads and subsequently sequenced library rgf reads was mapped to the FL contigs (6). Those clones whose 5'- and 3'-end reads map to the same contig were analyzed to determine sequence overlap (complete) or non-overlap (incomplete) (7). Only complete clones are considered, and a single representative of a clone is taken for each transcript resulting in 5,953 complete reference FLcDNAs (8).

Starting with a 434,384 *S. salar *EST assembly [33] (Figure 1 part 1), 81,398 contigs (Figure 1 part 2) were compared to the SwissProt protein database [51] (Figure 1 part 3) and 34,451 unique transcripts were identified. 14,021 of these were potential FLcDNA contigs as determined by similarity comparisons to known proteins (Figure 1 part 4). These assembled sequences represent potential full-length transcripts with significant similarity to SwissProt protein sequences. 10,026 (mean = 1,295 bp; range = 195 - 4,696 bp) of these contigs contained complete ORFs and 5,853 of these could be represented by a single completely characterized, non-redundant clone. These clone sequences are consistent with contig consensus sequences representing two or more different clones and were provisionally designated as reference FLcDNAs.

### *E. lucius *ESTs

A full-length biased, normalized cDNA library from *E. lucius *head kidney, spleen, heart and gill tissues was constructed and 15,360 clones were bi-directionally sequenced. 29,221 sequences averaging 731 bp were obtained and, with the previously available 3,612 EST sequences [33], assembled into 11,662 contigs (2,791 singletons and 8,871 clusters; mean cluster = 2.2 reads, 1,384 bp; max cluster = 106 reads). BLASTX analysis [52] revealed a total of 3,816 unique transcripts with strong SwissProt protein similarity (e-value ≤ 10^-5^). Using the same method outlined in Figure 1 part 4 for *S. salar*, 1,830 were identified as potential full-length transcripts. After ORF analysis (Figure 1 part 5), 1,543 FLcDNA contigs contained sequences corresponding to full-length proteins (mean = 1,044 bp; range = 312 - 2,984 bp) and 1,365 non-redundant reference clones were identified.

### Reference Full-Length cDNA identification using individual clone assembly

**Figure 2:**
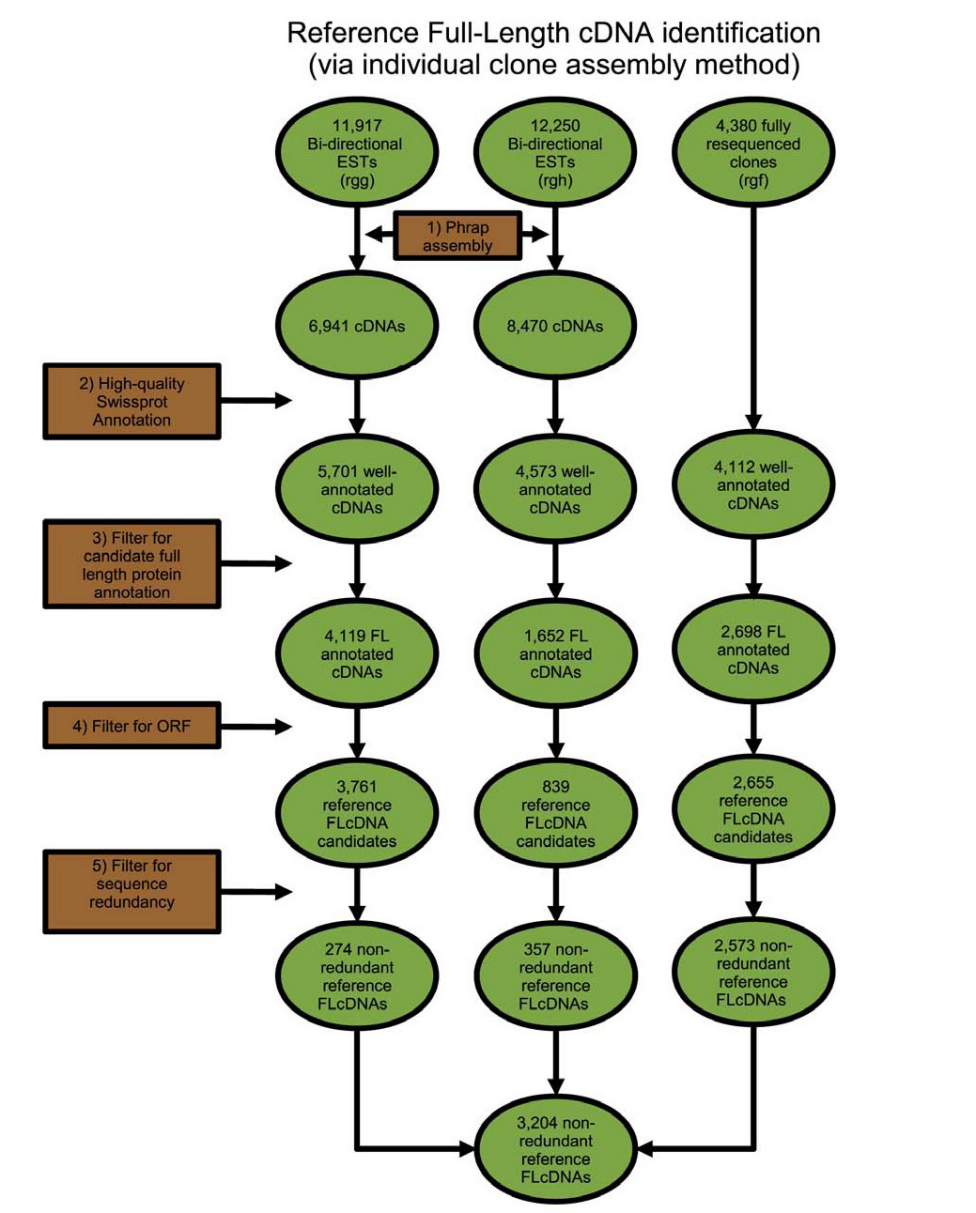
**Schematic of *S. salar *reference FLcDNA identification through individual clone assemblies**. Three full-length 5'-CAP enriched libraries were created. A 4,380 clone subset of library rgf was resequenced to completion. Libraries rgg and rgh were bi-directionally sequenced and individually assembled using PHRAP (1). A BLASTX was carried out resulting in a total of 14,384 well-annotated cDNAs (2), which were further reduced to 8,469 FL annotations by increasing the stringency of the local alignment length (3). In-frame annotation-flanking start and stop codons were found from the reduced set, resulting in a set of 7,255 reference FLcDNA candidates (4). Intra-library sequence redundancy was minimized using an all versus all pairwise BLASTN comparison (5), resulting in a total set of 3,204 non-redundant reference FLcDNAs.

Paired 5' and 3' sequence reads from short and mid-sized insert FLcDNA libraries from *S. salar *(rgg: 11,917 reads and rgh: 12,250 reads) were assembled individually to yield 6,941 rgg and 8,470 rgh cDNA clone sequences. These sequences were selected for further full-length, ORF and non-redundancy analysis (Figure 2). The short-insert library (rgg) yielded 274 new, full-length protein reference clone sequences. The midrange insert library (rgh) yielded 357 new FLcDNA reference clone sequences. The low yields of novel reference clones likely reflect similar clone insert sizes obtained from previous cDNA library characterizations.

The assembled 15,125 transcripts from the large-insert full-length cDNA library (rgf) from *S. salar *were initially examined for non-redundancy with existing full-length reference genes and potential for representing a full-length gene (5' and 3' non-overlapping clone sequences were consistent with the 5' and 3' ends of a known complete protein). Based on partial reference FLcDNA characterization, 4,380 clones were chosen for complete characterization using primer walking methods. Once these clones were completely sequenced, 4,112 were shown to have significant SwissProt similarity. Of those clones, 2,573 represented novel non-redundant *S. salar *transcripts with complete ORFs that corresponded to known proteins. Clones whose inserts contained full 5' annotation (Figure 2 part 3) and a proper ORF (Figure 2 part 4) were designated as reference FLcDNA clones. In total, 3,204 non-redundant reference FLcDNAs (Figure 2 part 5) were characterized from the three FLcDNA libraries of rgf, rgg, and rgh.

### Reference Full-Length cDNA assessment

**Figure 3:**
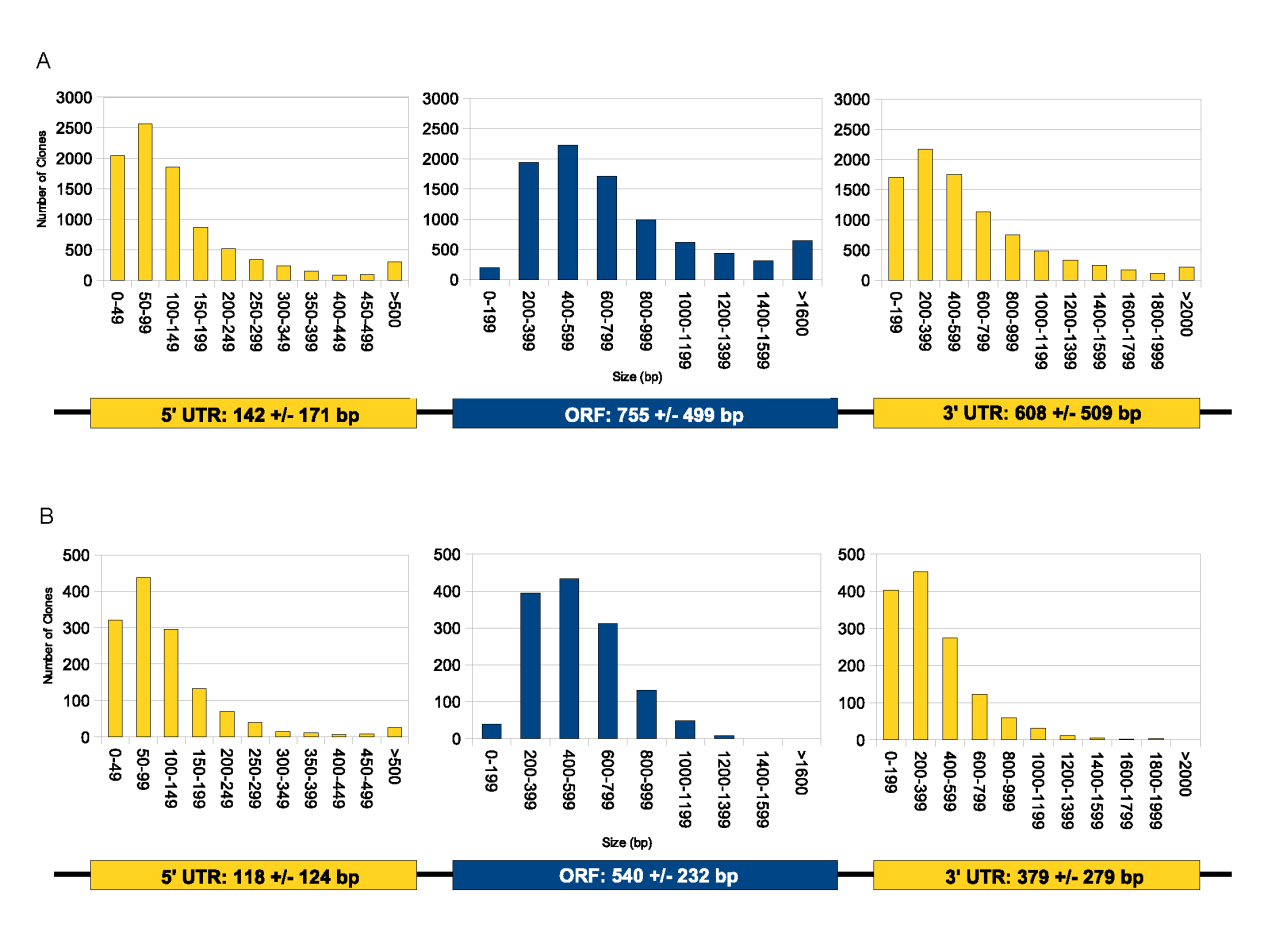
**Distributions and means of ORF, 5' and 3' UTR sizes in reference FLcDNAs for (A) *S. salar *(B) *E. lucius***. Each reference FLcDNA, determined by in-house annotation methods, was examined for an ORF, 5' UTR, and 3' UTR. Means for each region were calculated (+/- standard deviation). An ORF is characterized by a start (ATG) and an in-frame stop codon (TGA, TAG, TAA). The 5' UTR is calculated as the entire area upstream of the start codon, while the 3' UTR is considered the entire area downstream of the stop codon. Any 3' polyA tails were masked and were not included in UTR length calculations.

The 5' UTRs, ORFs and 3' UTRs for the 9,057 reference clones were characterized and the results are summarized in Figure 3. The mean reference FLcDNA length for 9,057 *S. salar *sequences (Figure 3a) is 1,450 +/- 794 bp (mean +/- SD), and ranges from 267 to 4,730 bp. Of these sequences the mean 5' UTR and 3' UTR is 142 +/- 171 bp and 608 +/- 509 bp, respectively. The mean reference ORF is 755 +/- 499 bp.

Similar analysis of *E. lucius *reference FLcDNAs (Figure 3b) shows a mean length of 1,003 +/- 286 bp, ranging from 312 to 1,731 bp. Mean UTRs in the 5' and 3' regions are 118 +/- 124 bp and 379 +/- 279 bp, respectively. The mean reference ORF is 540 +/- 232 bp.

The UTR results are comparable with efforts from groups which have compiled mRNA UTR databases. In one such study of UTRs for a variety of species belonging to the 'fish' category, these species were shown to have an average 5' UTR of 107 bp, while 3' UTRs averaged 397 bp [53]. These results indicate that the UTRs from this study are consistent with full-length sequences. While similar studies in this area are available for non-fish FLcDNAs, comparisons among more closely related organisms are lacking [54].

A contig confirmation and uniqueness study was performed on each reference FLcDNA sequence set and the results are outlined in Table 1. A comparison was done on each corresponding contig set in an attempt to demonstrate which reference FLcDNAs could be confirmed by an existing contig. Reference FLcDNAs from *S. salar *full-length libraries were not included in the contig assembly and therefore possibly contain novel cDNA inserts. There were 6,115 reference FLcDNAs that could be confirmed by a contig sequence for *S. salar*. All *E. lucius *clones were included in its contig assembly. As a result, the entire 1,365 reference FLcDNA set could therefore be confirmed by a contig sequence.

**Table 1:** Summary of confirmed and unique reference FLcDNAs in contig sets for *S. salar *and *E. lucius*

	Contigs	Reference FLcDNAs	Confirmed in Contigs	Unique
*S. salar*	81398	9057	6115	2942

*E. lucius*	11662	1365	1365	0

**Total**	93060	10422	7480	2942

Reference FLcDNAs were confirmed, using BLASTN, against their corresponding contig set. The remainder of the reference FLcDNAs are represented by clones that are similar to and consistent with SwissProt entries and are unique to the full-length libraries characterized in this study.

### *S. salar *and *E. lucius *alignments

FLcDNA transcripts from *S. salar *were used to identify protein coding regions for an analysis of silent and amino acid changing substitution rates in duplicated genes. The coding sequences were translated and used as queries in a TBLASTN comparison to the nucleotide database consisting of all *S. salar *and *E. lucius *EST assemblies. Contig sequences corresponding to the TBLASTN hits were organized into clusters, then translated, resulting in a cluster of nucleotide sequences and a corresponding cluster of protein translations for each full-length gene. A common region of alignment with respect to the translated ORF was found for the DNA sequences and the corresponding proteins based on length and quality of alignment criteria. A final screening process was performed to prevent allelic or distant homolog comparisons. 408 clusters contained the necessary one sequence from *E. lucius *and two sequences from *S. salar*. These sequences and alignments are given in Additional file 1.

Non-synonymous (d_N_), synonymous (d_S_) and ω (d_N_/d_S_) values were calculated for the 408 individual gene trees to investigate patterns of evolution. A value for ω that is < 1 over the alignment is indicative of purifying selection (the rate of amino acid changing substitutions is less than the rate of incorporation of synonymous mutations). A value for ω that is > 1 is indicative of diversifying or positive selection [55].

### Pairwise comparisons to determine d_S _and ω

**Figure 4:**
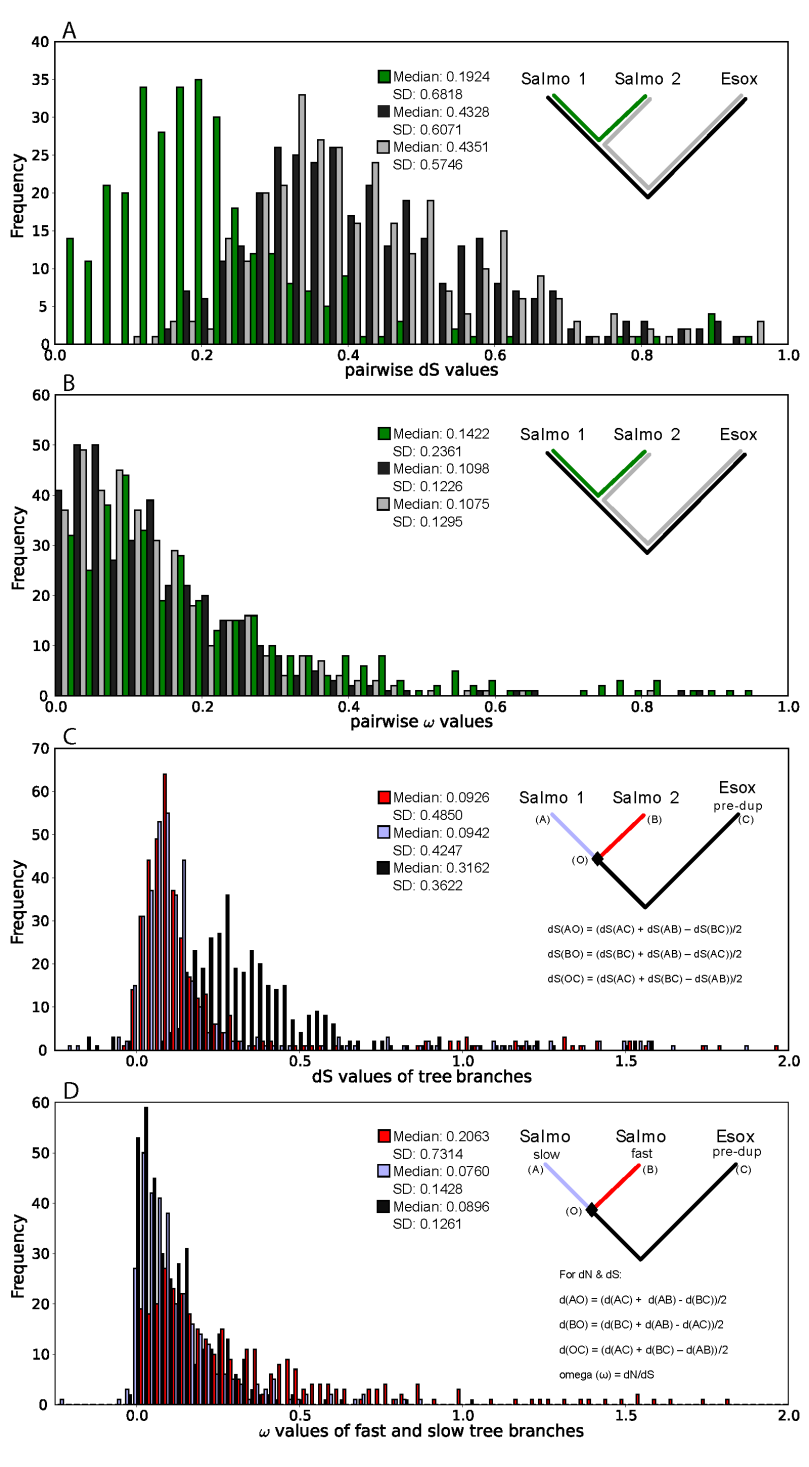
**Frequencies of d_S _and ω values for comparisons within *S. salar *and *E. lucius *gene trios**. (A) Distributions of d_S _values from pairwise comparisons within gene trios: between *S. salar *paralogs (green) and between each of the two *S. salar *paralogs and its corresponding *E. lucius *ortholog (gray and black). (B) Distributions of d_N_/d_S _ratios (ω) from pairwise comparisons within gene trios: between *S. salar *paralogs (green) and between each of the two *S. salar *paralogs and its corresponding *E. lucius *ortholog (gray and black). (C) Distributions of d_S _values separated into individual tree branches based on gene trios. Values from pairwise comparisons were used to calculate silent substitution rates for periods before and after the salmonid tetraploidization event. The light blue curve represents frequencies of d_S _values from the duplication event to one *S. salar *paralog, the red curve from the duplication event to the other paralog, and the black curve prior to the genome duplication to the *E. lucius *ortholog. (D) Distributions of d_N_/d_S _ratios separated into branches where one *S. salar *paralog, that which has the lower ω value, is considered to be a *slow *branch (light blue curve) and the other paralog (red curve) is considered to be more quickly diverging (*fast *branch for the purposes of labelling). The black curve displays frequencies of ω values between the *E. lucius *ortholog and the genome duplication.

For each gene cluster, the corrected number of synonymous substitutions per synonymous site (d_S_) was determined by comparing the *E. lucius *gene to each of the duplicate *S. salar *genes (gray and black lines) and the *S. salar *duplicate genes to each other (green line; Figure 4a). For each branch, the frequency of d_S _values is plotted in Figure 4a. *S. salar *gene duplicate d_S _values (green lines) have a median value of 0.192 and the *E. lucius *to *S. salar *d_S _values (gray and black lines) have a median value of 0.434. This difference confirms that the salmonid genome duplication occurred more recently than the separation of *E. lucius *and *S. salar *lineages.

The ratios of non-synonymous to synonymous substitution rates, or ω values, were in a similar manner calculated for each gene set and the frequency of ω values presented in Figure 4b. The median ω for *E. lucius *to each of the duplicate *S. salar *genes (gray and black lines) is 0.109 and the median ω for the duplicate *S. salar *genes (green line) is 0.142. The low ω values for all three sets of pairwise comparisons indicates an average of 7-9 synonymous substitutions for every non-synonymous substitution. This ratio confirms that purifying selection is the predominant evolutionary force in these genes. In most cases, both copies of these genes appear to have had their original functions retained based on the relatively low ratio of substitutions and high similarity between sequences. It is worth noting however, that poorly aligning and therefore potentially more divergent regions are trimmed from the overall alignments. Therefore, these estimates may be on the conservative side.

The paralogous comparisons (green line) produced ω values that were generally larger than the orthologous comparisons (gray and black lines). Upon using a Kruskal-Wallis test to compare distributions, both sets of orthologous comparisons were found to be significantly different from the paralogous comparisons (p-value = 1.671 × 10^-5 ^and 2.359 × 10^-5^) while orthologous sets were not significantly different with respect to each other (p-value = 0.9188). Therefore, while there is a large variance in the level of selection among the different genes, this result supports a small but significant relaxation in the level of selection pressure following gene duplication. This result is consistent with a comparison of 445 gene duplicates in the polyploid *Xenopus laevis *[37].

### d_S _and ω for tree segments

To more closely examine the effects of evolutionary pressures before and after duplication of the salmonid genome (represented by a diamond in the accompanying tree diagram), the substitution rates and ratios were separated into three tree segments (shown in Figure 4c, 4d). This subdivision was accomplished by using all three pairwise comparisons to calculate d_S _values and ω values from the occurrence of the genome duplication (point O) to each extant gene (points A, B, and C).

This calculation provides an approximation for the number of substitutions before and after the duplication event. The post-duplication branches (in red and light blue) yielded median d_S _values of 0.0926 and 0.0942 and the pre-duplication branch yielded a median d_s _value of 0.3162. *E. lucius *sequences clearly diverge from the *S. salar *paralogs to a much greater extent than the *S. salar *paralogs diverge from each other. This again is consistent with the WGD occurring in salmonids but not in ecosids. The few values that are less than zero are presumably a result of the variation in the divergence of the three sequences with respect to one another and chance convergent substitutions.

To pursue the observation in Figure 4b showing a relaxation of selection following gene duplication (green line) and operating under the assumption that one duplicate could be retained and preserve its original function, freeing the other to diverge [56], the two post-duplicate branches were separated into two groups; one member of each pair represented the *slow *branch (i.e. the branch with the lower ω, indicating more purifying selection) and the other member represented the *fast *branch (i.e. with the higher ω, indicating possibly relaxed selection). While this method of sorting duplicate genes is somewhat arbitrary, the results can be reviewed with respect to an ancestral branch leading to *E. lucius*. Selection values (ω) for the fast and slow duplicates along with the ancestral branch were calculated for each gene set and the frequency of ω values plotted in Figure 4d. In the slow *S. salar *branch (light blue) the median ω was 0.0760 and in the ancestral branch (black) the median ω was 0.0896. While the slow branch and pre-duplication branch do differ significantly from each other in terms of the means of the ranks of the data (Kruskal-Wallis test, P = 0.00623), the slow branch has a very similar median ω to the pre-duplication condition. The fast branch, on the other hand, has a much higher average ω (red curve, median = 0.2063) than both of the other branches. These results are consistent with the view that after the WGD there was little change in the evolutionary rate for one member of the pair; though in many cases, the rate of incorporation of non-synonymous changes increased dramatically for the other member. Despite a high level of variation, these data suggest that there is some asymmetry in evolutionary pressures on paralogs.

### Gene Ontology analysis

In order to determine if there were ontological categories that were enriched for gene pairs that were subjected to more asymmetrical rates of evolution than others, Gene Ontology terms [57] were found for two groups of gene sets: those that had high fold differences (> 3x; n = 67) in the ω values found for the two *S. salar *branches and those that had low or no fold differences (<1.75x; n = 61). The results are shown in Table 2. For the most part, the two groups are populated by categories with similar proportions. A few notable exceptions include a larger proportion of genes involved in nucleic acid metabolic processes (GO:0006139) in the high fold-change group relative to the low. Likewise, the low fold-change group has a higher proportion of "other" metabolic processes (various IDs) such as lipid and carbohydrate metabolism. However, the relatively low number of genes in most categories limits the possibilities in this study of correlating ontological terms with specific patterns of evolution.

**Table 2:** Proportions of genes in GO categories

Categories	ID	High fold-changes		Low fold-changes	
localization (transport, cell motion)	GO:0051179	11	16.4%	10	16.4%

nucleic acid metabolism	GO:0006139	10	14.9%	3	4.9%

protein metabolism	GO:0019538	6	9.0%	6	9.8%

other metabolic process	Multiple IDs	8	11.9%	16	26.2%

development	GO:0032502	6	9.0%	5	8.2%

translation	GO:0006142	6	9.0%	4	6.6%

transcription	GO:0006350	5	7.5%	3	4.9%

apoptosis	GO:0006915	3	4.5%	5	8.2%

response to stimulus	GO:0050896	4	6.0%	2	3.3%

cell proliferation	GO:0008283	3	4.5%	2	3.3%

cell cycle	GO:0007049	3	4.5%	2	3.3%

signal transduction	GO:0007165	2	3.0%	3	4.9%

**Total**		67	100%	61	100%

Gene Ontology terms are given for gene trios in which there is a high fold-change in ω between paralogs (asymmetrically evolving duplicates) or a low fold-change in ω (symmetrically evolving duplicates). The proportions of genes contained within Gene Ontology categories are compared between the two groups. Nucleic acid metabolism (GO:0006139) is more highly represented in asymmetrically evolving gene duplicates.

## Discussion

The objectives of this study were to: 1) characterize a large unambiguous set of reference gene sequences to compare with alleles and duplicates in *S. salar*, genes in other salmonid species, and genes in more distantly related fish species; 2) expand genomic resources for a representative member of the closest non-tetraploidized fish group (Esociformes: *E. lucius) *to provide a reference for the study of WGD in salmonids; and 3) identify patterns of change in the evolution of duplicated genes in the autotetraploid *S. salar*.

Genome duplications have a profound impact on the physiology, reproductive biology, ecology and evolution of a species. Salmonids (11 genera and 66 species) [1] are one of the most economically important and most studied groups of fish. A purported WGD in their common ancestor between 25-100 million years ago [35], after the separation from esocids, plays a prominent role in understanding the biology of this group. The different salmonid species are currently in the process of reverting to a stable diploid state through deletions and rearrangements [17,32-34]. In the absence of a completed genome, there is a significant problem in distinguishing among numerous duplicates, alleles and other very similar sequences. The integrity of genes resulting from assembling large numbers of partial mRNA sequences (ESTs) remains open to question. To resolve this problem, a collection of reference genes containing CDS and flanking UTRs coming from single, completely characterized cDNA clones provides an essential resource in gene identification, future genome annotation, and the study of evolutionary patterns.

An analysis of existing EST data from *S. salar *led to estimates of 10,026 FLcDNA contig consensus sequences. However, these contigs may represent an amalgamation of many unique transcript products with high similarity, rather than a single unique allele. 9,057 *S. salar *reference FLcDNA clones were determined in this study to resolve this issue. These FLcDNA sequences represent a significant community resource, adding to the current knowledge base on salmonid biology. These sequences can also serve as scaffolding with which to aid in genomic sequencing and creation of physical maps in other salmonids. The increasing popularity of microarrays in gene expression experiments has allowed for more precise control of probe design and information on full-length sequences enables probes to be optimally designed with higher specificity. An increase in probe-binding specificity reduces unwanted cross-species interactions. Salmonid research benefits from a fuller characterization of *S. salar *genes.

To expand evolutionary studies of salmonids, 29,221 *E. lucius *ESTs were obtained and when combined with the existing 3,612 EST sequences (32,833 total), 11,662 contigs and 1,365 FLcDNA reference sequences were identified. This resource not only provides an important initial genetic foundation for the study of pike throughout North America, Europe and Asia, but also provides essential information on a diploid reference species for the study of WGD in salmonids.

In this study, the FLcDNA sequences from *S. salar *along with homologous data from *E. lucius *were used to analyze evolutionary trends in some of the genes in the pseudotetraploid genome of *S. salar*. While the salmon genome may still be in the process of returning to a stable diploid state, it is evident that many gene duplicates have been retained. The peak in Figure 4c indicates a collection of genes that arose from a duplication event after the separation of esocids from a salmonid ancestor. These genes are likely to be still active because the data for this study are based on mRNA, EST-derived sequences.

It is interesting to note that both Morin et al. [37] and the present study started with approximately 10,000 full-length transcripts. By selecting a subset of sequence clusters that fulfil alignment and homology criteria, both studies ended up with only 400-450 gene sets. This ~4% yield is due in part to the strict criteria for usable sequences as well as the more limited *E. lucius *dataset from which to draw sequences. Further investigation should be undertaken to determine if both *X. laevis *and *S. salar *have retained similar proportions of gene duplicates, which would be of great interest in understanding responses to tetraploidization events. The numerous other species in the salmonid family provide an opportunity to facilitate finer analysis of the genome duplication, once additional data have been gathered for them. Moreover, the additional gene sets that contained more than two *S. salar *sequences in addition to the *E. lucius *ortholog (approximately 300 sets) could be studied to gain an understanding of some of the smaller scale duplication events or potentially more ancestral WGDs.

Over the last century, many individuals and groups have developed ideas about gene duplication in evolution and its importance in expanding on existing biological functions [58]. A central model that has been strongly supported by Ohno [56] states that a duplicate gene can accumulate mutations and become non-functional (non-functionalization) or diverge to a novel function (neo-functionalization) while the other duplicate keeps its original function. Other models have been proposed including sub-functionalization [59,60], where both duplicates accumulate mutations resulting in complementary expression, leaving each copy with its own sub-function. It is of interest to look for signatures of different types of selection in order to better understand models that may be directing the fates of one or both paralogs.

Based on the observation that the genes under investigation are in fact being transcribed to some degree in *S. salar*, it would be expected that purifying selection would be acting on both duplicates. The vast majority of ω values that are presented (Figure 4) are much less than one. It is apparent that negative selection is the predominant force in this evolutionary process as was also found in the similar analysis done by Morin et al. [37]. However, relative to the state of the genes before the duplication, there is significant relaxation of selective pressure on at least some of the paralogs, suggesting reduced constraints. This relaxation is consistent with the idea that having redundancy in the genome will result in increased freedom for divergence [56,58]. These trends could facilitate neo-functionalization or modification of existing functionality taking place in some of the paralogs.

Data in this study provide evidence that selection constraints are not acting on both gene duplicates to the same extent. In a number of the 408 gene sets examined, one paralog may be relaxed, while it appears that the other is maintained to roughly the same degree as the pre-duplication single-copy gene. This asymmetrical pattern of evolution has recently been observed in specific *Hox *clusters in *S. salar *[61] as well as in earlier genome-wide studies in other organisms such as *Drosophila melanogaster *and *Caenorhabditis elegans *[39].

The question that results from this observation centers around the fate of these duplicate genes that are operating under relaxed selection. The paralogs that were studied are still being transcribed and are presumably functional (with the possible exception of some rarely transcribed pseudogenes) and have not been subject to non-functionalization. Duplicates that were deleted since the WGD would not be observed and neither would the presumably large number of duplicates that have become pseudogenes. Conclusive evidence for a general trend of positive selection was not found for the set of genes, since nearly all ω values were much less than one, though there were a few higher post-duplication values that suggested some duplicates may have been influenced by directional selection. The few genes that did have an ω value greater than one showed no enrichment for an ontological category (data not shown). Turunen et al. [40] looked at asymmetrically evolving gene duplicates in yeast and found evidence for relaxation of selective pressure, sub-functionalization, and even neo-functionalization, though the average ω was significantly less than one. Therefore, it is not surprising that a strong signature of diversifying selection was not detected. Positive selection that may have occurred over a small region or short period of time could be masked by a larger overall pattern of negative selection. For example, once a neo-functionalization event has occurred, purifying selection would act to maintain that new function in the long term. Indeed, Hughes et al. [62] reported 30-50 million years of divergence to be the upper limit of detection of positive selection in eukaryotes using d_N_/d_S _analysis. Looking at a variety of salmonid species in a comparative fashion could enable a higher resolution study of changes in evolutionary pressures and may provide more clues as to the events that took place in the duplicated genes soon after the tetraploidization. In addition, other groups [63,64] have studied polyploidization in *Xenopus *species using some alternative methods that may be applicable to *S. salar *in future efforts. One example was using transversion rates at four-fold synonymous codon positions (4 DTv) to measure evolutionary divergence, though saturation of mutations at synonymous sites was not a problem for the present study.

Functional gene groups defined by Gene Ontology terms were found for *S. salar *gene duplicates that displayed either substantial or very small to no differences in selection constraints (i.e. evolving asymmetrically or symmetrically, respectively). The proportions of genes falling into the defined categories were generally quite similar (Table 2). However, one interesting result was the higher percentage of genes involved in nucleic acid metabolic processes (GO:0006139) (e.g. RNA processing and DNA metabolism) in the group of gene sets in which a large difference in selection constraints was identified. In this case, the conclusion that nucleic acid metabolism genes were more often present in the asymmetrical group than the symmetrical group would be consistent with earlier studies, which found that nucleic acid processing and nucleoside metabolism functional groups were selectively lost after whole genome duplications in *X. laevis *and *A. thaliana*. This suggests that nucleic acid processing and nucleoside metabolism functional groups of genes may have a greater chance of conferring dosage sensitivity [37,38].

## Conclusions

During the rediploidization after a WGD in the common ancestor of the salmonids, many gene duplicates were retained. There is strong evidence that purifying selection is the predominant force acting on these gene duplicates. However, there is also evidence that this selection has been relaxed significantly in genes after the duplication. Furthermore, the relaxation of selection occurred in an asymmetrical manner, preferentially allowing the divergence of one duplicate over the other. Though more research is needed to gain a higher resolution picture of the fates of the retained duplicates, these results add to the body of knowledge surrounding models of evolution following genome duplications and shed more light on the complex salmonid genome.

## Methods

The sequence data from this study have been submitted to GenBank's dbEST [65] and core nucleotide databases under search terms 'salmo salar [orgn] AND leong' and 'esox lucius [orgn] AND leong' for *S. salar *and *E. lucius*, respectively. The corresponding author may also be contacted for GenBank accession numbers.

### Tissues, RNA, and Sampling

Adult *S. salar *tissues (brain, kidney, spleen) were obtained from Robert Devlin at the Department of Fisheries and Oceans (WestVan Lab, West Vancouver, British Columbia). Adult *E. lucius *tissues (head kidney, spleen, heart, gill) were obtained from Frank Koop at Charlie Lake (Fort St. John, British Columbia). Tissues were rapidly dissected, flash-frozen in liquid nitrogen or dry ice, and stored at -80°C until RNA extraction.

### cDNA Libraries

Three full-length, non-normalized cDNA libraries were constructed using a full-length cDNA library protocol (Research Genetics Inc.). This protocol employed an enrichment of 5'-CAPed mRNA which prevents truncated mRNA from being reverse-transcribed, followed by transfer of intact double-stranded cDNAs directly into the library vector using Gateway^® ^recombination cloning. An estimated 65-85% of the clones were full-length [50].

Different mRNA size fractions were used in the construction of the three libraries. The libraries were created using transcripts between 0.6 to 1.1 kb (rgg), 1.1 to 2.0 kb (rgh) and > 2.2 kb (rgf). The cDNA libraries were directionally constructed (5' M13 Forward, 3' M13 Reverse) in pENTR222 vector (Research Genetics Inc.).

The *E. lucius *library (evq) was made from head kidney, spleen, heart and gill cDNAs that were normalized and directionally cloned (5' M13 Forward, 3' SP6) in pAL17.3 vector (Evrogen Co.). Sequences from a previously characterized *E. lucius *brain, kidney, and spleen library [33] were also utilized.

### Sequencing, Sequence Analysis, and Contig Assembly

Clone libraries were plated and robotically arrayed in 384-well plates as detailed previously [33]. Plasmid DNAs were extracted and BigDye Terminator (ABI) cycle sequenced on ABI 3730 sequencers using conventional procedures and the following primers: 5'-T18-3', M13 forward (5'-GTAAAACGACGGCCAGT-3'), M13 reverse (5'-AACAGCTATGACCAT-3' or 5'-CAGGAAACAGCTATGAC-3'), and SP6WAN (5'-ATTTAGGTGACACTATAG-3') for 3' end sequencing of Evrogen libraries. Base-calling was performed using PHRED [66,67] on chromatogram traces. Vector, polyA tails, and low quality regions were trimmed from EST sequences. Short (100 bp) low quality sequences were discarded. Assembly of *S. salar *ESTs into contigs employed two-stage processing using PHRAP (Figure 1 parts 1-2) [33]. CAP3 [68], using default parameters, was employed for a single assembly of *E. lucius *ESTs in place of the PHRAP two-stage approach, the purpose of which is to handle WGD transcriptomes.

### FLcDNA contig identification

The analysis of full-length transcripts began with all EST contig sequences. Since each contig represents a potential transcript, it must be determined if a transcript is complete or incomplete. A complete or full-length transcript contains an entire CDS for a gene product, along with the flanking 5' and 3' UTR. Incomplete transcripts are mRNA that have not been fully reverse-transcribed during cDNA library creation, and therefore may not contain the complete CDS or the 5' UTR. Because of the selection for polyA tails during cDNA library creation, both incomplete and complete transcripts contain a polyA tail. Inherent experimental errors in the reverse transcription step during cloning result in 5' incomplete cDNA inserts.

Using an e-value filter of e ≤ 10^-5^, the top ten SwissProt high-scoring segment pairs (HSPs) from BLASTX for each contig were analyzed in succession to identify the correct open reading frame (Figure 1 part 3). Full database protein matches must be contained within a full-length transcript sequence. HSPs often do not match a homologous protein in its entirety. This situation exists for the following reasons: i) a transcript is incomplete; ii) a transcript represents a pseudogene; iii) a transcript represents a novel gene product, but contains a domain common to an existing non-homologous protein. In cases where the match region between a transcript query and a subject protein sequence does not fully encompass the length of the subject protein, the two complete sequences are checked to determine whether the 5' end of the transcript extends beyond the 5' end of the known database reference protein sequence. In situations where the transcript is not long enough to accommodate the full database protein length, transcripts are disregarded from further FLcDNA consideration (Figure 1 part 4). In cases where the transcript is long enough to contain the known database reference protein, the transcript is kept for further analysis.

An ORF is a single continuous region on a processed transcript sequence that encodes a complete protein. These regions are defined by a start codon (ATG) and end with an in-frame (non-coding) stop codon (TAG, TAA, or TGA). When a potential start codon is identified, a corresponding in-frame stop codon is verified to complete an ORF. Stop codons found upstream of the start are useful but not essential in defining the proper coding region. Start codon positions are determined by examination of ATG motifs present upstream, in-frame or within 30 bp downstream of the beginning of the aligned reference protein. Coding regions often contain multiple methionine codons, which may obscure prediction of a start codon. If a methionine codon is not found between the first upstream stop codon and the predicted start codon, it is assumed that the start codon is correct. If a methionine is found upstream of the predicted start codon and still is in-frame with the downstream stop codon, this new ATG motif position is assigned as the correct start codon. Once a start codon is identified, a corresponding in-frame stop codon is verified to form the completed ORF (Figure 1 part 5).

### Reference FLcDNA identification

Complete transcripts whose coding regions can be fully represented by a single cDNA clone sequence are considered reference FLcDNAs. These FLcDNAs contain 5' and 3' UTRs flanking an ORF that matches or is consistent with a known protein identified by a BLASTX similarity search.

Subsequent to the initial clustering and annotation of 434,384 ESTs to establish the putative transcript set, three full-length cap-trapped libraries (rgg, rgh, rgf) were created and bi-directionally sequenced. Of these libraries, rgf ESTs were assembled, using PHRAP, to produce transcripts to be compared to the established set of 81,398 putative transcripts [33]. The clone reads from the original libraries that were used to produce the putative transcript set were mapped back, via local alignment, to this putative set to determine which clones contained a reference FLcDNA insert. Library rgf was also mapped back to the putative transcript set. Reads from identical clones that map against the same putative transcript and contain sequence overlap are considered to be from a reference clone. If the forward and reverse reads from the same clone both overlap an identical region of the transcript, that clone is classified as being complete. There are cases where clones have forward and reverse reads that do not overlap when mapped to the same transcript. In this scenario, a gap exists between the reads when mapped to the cluster, suggesting an area for which primers can be designed for further sequencing. These clones are known as incomplete clones, and formed a subset of 4,380 rgf clones that were later resequenced to completion. Libraries rgg and rgh were not included in any of these comparisons but were analyzed on an individual clone basis (discussed below).

The 81,398 putative transcripts were established using a two-stage EST clustering process [33]. As a result, the second-stage assembly begins with sequences from the first-stage assembly. Prior to assembly, gaps from the sequence set need to be removed. As a result of a two-stage assembly, not only does one lose gaps that initially may have been introduced, but EST read names are also lost. The modification of gaps in assembled sequences affects the positions in the reads the assemblies are composed of. To recalculate read positions and reference FLcDNA clones, a local alignment of all reads from all libraries (except rgg, rgh) was performed against the putative second-stage transcript set of 81,398 sequences. Reads from identical clones that map against the same transcript set corresponding to FLcDNA contigs, regardless of sequence overlap, are determined (Figure 1 part 6).

All 6,081 complete (overlapping reads) clones (Figure 1 part 7) that flanked the entire predicted ORF region, in the set of 10,026 FLcDNAs, are selected and form the reference FLcDNA clone set. In this set, more than one complete reference clone may map to a single transcript. Therefore, to produce a non-redundant set of complete FLcDNA reference clones, only the longest complete reference clone that maps to a specific transcript is selected. In the case where clones are of equal length, the clones are simply chosen according to alphabetical order, resulting in 5,853 non-redundant reference clones that are unique to a single transcript (Figure 1 part 8).

### Reference FLcDNA identification using individual clone assembly

In addition to analyzing reference FLcDNA clones via transcript mapping, two full-length libraries (rgg, rgh) and a single fully sequenced full-length library subset (incomplete clones from rgf) were examined. Each of these three *S. salar *libraries was analyzed independently.

Clones were assembled individually so that reads that were already known to be from the same clone could be explicitly allowed to join, while erroneous additions of other sequences could be minimized. Using this method, libraries rgg, rgh, and a portion of rgf clones that were selected to be resequenced were analyzed independently from each other. For all sequence reads from rgg and rgh libraries, individual clone PHRAP assemblies [69] (minscore 8, repeat stringency 99%) were performed (Figure 2 part 1).

The subset of 4,380 selected rgf library clones were fully resequenced (minimum PHRED 20 for entire sequence) [66,67]. Those clones that contained a gap or the end sequences were of poor quality were rearrayed to a 384-well plate for further finishing via primer-walking. All sequences from this fully-sequenced group could therefore be directly selected for further full-length analysis.

Redundancy was minimized by performing an all versus all pairwise BLASTN comparison per library. Transcripts that showed greater or equal to 98% similarity over 200 bp were considered redundant. For sets of redundant transcripts, the longest sequence was taken as the non-redundant representative (Figure 2 part 5).

### Reference FLcDNA assessment

To properly assess reference FLcDNAs, sequences were checked for polyA tails. A polyA tail is defined as a 3' region of 15 or more consecutive "A" resides. If such a polyA tail was detected, those sequences were deleted as well as all subsequent downstream sequence.

For *S. salar *and *E. lucius*, reference FLcDNAs that could be confirmed by a contig sequence were identified. Using BLASTN to determine matches, each reference FLcDNA set was compared to its contig assembly. Reference FLcDNAs that showed 100% similarity over ≥ 95% of its sequence were considered to be identical. Those that did not possess confirmed identity were categorized as unique reference FLcDNAs.

### Selection of homologous genes

The 10,026 full-length *S. salar *cDNA contigs were used to identify homologous sequences and construct sets containing two paralogs from *S. salar *and one ortholog from *E. lucius *for determination of synonymous and non-synonymous substitution rates. It was necessary to start with known full-length contigs in order to be certain of the translation frame and ORF in the *E. lucius *and *S. salar *ESTs. Full-length sequences with the same accession number as another were removed from the query set resulting in a set of 5,219 unique contigs. This was because sequences with the same annotation would be likely to return the same cluster of ESTs when used to identify homologous sequences. The full-length sequences were translated to protein using ORF information. A TBLASTN was performed using these amino acid sequences as queries against a translated nucleotide database consisting of all of the *S. salar *and *E. lucius *EST contig assemblies, 93,060 in total. An e-value of 10^-10 ^or less was required for a match and 100 matches for each query were considered. The contigs corresponding to the BLAST matches were gathered into clusters, one cluster for each query sequence. As a preliminary screening function, the BLAST alignment was checked for percent coverage of the length of the amino acid query sequence. If the alignment covered 50% or greater, it was put into the cluster; otherwise, the alignment was discarded. BLAST information (hit region, frame of translation, and percent positive and identical matches) for each hit was retained. Each group of contigs was then translated using the frame information from the TBLASTN results and the resulting amino acid sequences were put into another cluster. Thus two corresponding sets of clusters were created, one protein and one nucleotide.

### Determination of alignment regions

The DNA sequences in each individual cluster were trimmed to a common region of alignment with respect to the query protein sequence. The sequence that had the longest local alignment was compared with the sequence with the next longest alignment, and the common aligned region was retained, potentially trimming one or both ends of either sequence. This was repeated with sequences having shorter and shorter alignments until a common region was found for that cluster. The minimum length of the alignment was 300 bp; if a sequence's alignment would cause the common region to drop below 300 bp, that contig was removed entirely. In addition, the original TBLASTN alignment was required to have at least 75% positive amino acid matches. This same process was done on the protein sequences to get the same alignment regions using 100 residues as the minimum length.

### Sequence alignment

The trimmed protein sequences were aligned using ClustalW with default parameters [70]. Using the ClustalW alignments and the nucleotide clusters, RevTrans was used to create codon-aware DNA alignments [71]. The alignments were further screened for the presence of alleles and very similar sequences as well as odd sequences that did not closely match the cluster. This filtering was done by aligning each sequence in the cluster with every other sequence. If an alignment showed greater than 98% identity or less than 60% identity or the alignment was shorter than 90% of the length of the longer sequence, the sequence was dropped from the cluster.

### d_N_/d_S_ estimation

Only the final alignments containing one sequence from *E. lucius *and two sequences from *S. salar *were used in the analysis, 408 in total. The 408 clusters with the required three sequences were then converted from FASTA format to a sequential alignment form that the PAML package could use as input. The YN00 program in the PAML package was used with default parameters on each gene trio to determine d_N _and d_S _rates [72]. In addition, ω (d_N_/d_S_) values for the individual branches of the tree were estimated based on the formulae(1)(2)(3)

where A and B are the extant paralogs, C is the extant ortholog, and O is the point of gene duplication [73].

### Gene Ontology analysis

Gene Ontology terms were found for the sequences that had the highest fold-change in ω between the post-duplication branches (> 3x; n = 67) as well as the lowest fold-changes (<1.75x; n = 61). BLASTX searches [74] were performed on sequences against the SwissProt database [51]. Gene Ontology terms were taken from Entrez Gene [75] for the top hit using e ≤ 10^-10^.

## Authors' contributions

JSL and SGJ carried out the *in silico *analyses and drafted the manuscript. KRvS, GAC, and AMM coordinated design, preparation and sequencing of libraries. NYL, SM, RM, SJMJ, and RAH performed large-scale sequencing. BFK and WSD conceived of the study, participated in its design and coordination, and helped to draft the manuscript. All authors read and approved the final manuscript.

## Supplementary Material

Additional file 1 **Sequence data for gene trios**. Sequence data for all gene trios used in the analysis are provided in FASTA format as a text file named gene_trio_seqs.txt. Each gene trio contains three nucleotide sequences from *E. lucius *and *S. salar *with a header containing species identifier and a unique contig number (e.g. >eluc_5216861). Sequences are aligned and can be directly translated. Click here for file
